# From dark to darkness, negative phototropism influences the support-tree location of the massive woody climber *Hydrangea serratifolia* (Hydrangeaceae) in a Chilean temperate rainforest

**DOI:** 10.1080/15592324.2022.2122244

**Published:** 2022-12-07

**Authors:** W. David Rodriguez-Quintero, María Moreno-Chacón, Fernando Carrasco-Urra, Alfredo Saldaña

**Affiliations:** a Departamento de Botánica, Universidad de Concepción, Concepción, Chile; b Centro de Ecología Aplicada Ltda, Principe de Gales 6465 La Reina, Chile; c Instituto de Ciencias Biológicas, Universidad de Talca, Talca, Chile

**Keywords:** support-tree location, woody root climber, Hydrangeaceae, temperate rainforest, circular statistics, negative phototropism

## Abstract

Climbing plants rely on suitable support to provide the light conditions they require in the canopy. Negative phototropism is a directional search behavior proposed to detect a support-tree, which indicates growth or movement away from light, based on light attenuation. In a Chilean temperate rainforest, we addressed whether the massive woody climber *Hydrangea serratifolia* (H. et A.) F. Phil (Hydrangeaceae) presents a support-tree location pattern influenced by light availability. We analyzed direction and light received in two groups of juvenile shoots: searching shoots (SS), with plagiotropic (creeping) growth vs. ascending shoots (AS), with orthotropic growth. We found that, in accordance with light attenuation, SS and AS used directional orientation to search and then ascend host trees. The light available to *H. serratifolia* searching shoots was less than that of the general forest understory; the directional orientation in both groups showed a significant deviation from a random distribution, with no circular statistical difference between them. Circular-linear regression indicated a relationship between directional orientations and light availability. Negative phototropism encodes the light environment’s heterogeneous spatial and temporal information, guiding the shoot apex to the most shaded part of the support-tree base, the climbing start point.

## Introduction

Plants are not as passive as they appear, experiencing motion through timescales and distances not always apparent to human observers.^1–3^ They actively forage for limited abiotic and biotic resources distributed in unequal quantity, quality, space, and time.^4–6^ Foraging is an iterative process^7^ based on searching (detecting resources), encountering (growth toward a potential resource) and decision-making (assessing whether it is worth the effort to obtain a resource).^8–13^ This process is crucial and effective in young plants because it is directly related to the present and future competitive environment that they will experience through their ontogeny.^14–19^

In this context, plant tropisms, active responses involving acquiring and processing information from directional physical stimuli or environmental cues–coupled with plant modular structure–guide individuals toward favorable conditions.^10,20–27^ Light performs a dual role in plants as the fundamental resource for photosynthesis and provides external environmental information (cues) as directional stimuli.^25,28–32^ When light availability is heterogeneous, plant shoots change their directional growth pattern (reorient) toward the optimal light intensity; they then assess the foraging efficiency of obtaining the light and resources associated with light cues.^11,32–43^

Climbing plants rely on their attachment to suitable external supports through their vegetative structures and directional searching movements.^33,35,38–40,44–50^ This group of plants also present a life-history strategy of extensive clonal growth, a principal driver that determines their local spatial distribution patterns, regeneration, colonization success, and contributes to their abundance and resources foraging.^51–55^ Clonal plants can employ as response to light competition in three categories, considered as vertical (confrontational) growth, shade tolerance or lateral avoidance.^13^ Individuals successfully attached to a support-tree improve their light intake, are more abundant and present higher biomass, physiological yield, reproductive output, and lower herbivory than those unattached.^12,47,56–61^

In woody climbers with adventitious roots, the plagiotropic (creeping) shoots and seedlings exposed to bright light, or even to low-light intensity, grew toward dark sites and moved away from light, exhibiting *negative phototropism*.^33,35,38–40,42^ In the chiaroscuro of the forest floor, potential support-trees have been found in the darkest sectors,^33^ and under shady conditions, the climbing habit has also been found to be enhanced.^51,56,58,59,62^ Nevertheless, how the creeping shoots of woody climbers reach and ascend support-trees, thanks to an efficient search behavior, has received little research attention,^33,35,42,51,63,64^ compared to studies regarding mechanisms that explain property components such as density, biomass, diversity or distribution of climbing plants.^53,60,65–72^

In a southern Chilean temperate rainforest, the density of climbers was 66 individuals per 0.1 ha, with the native and woody root climber (liana) *Hydrangea serratifolia* (H. et A.) F. Phil (Hydrangeaceae) being the dominant species, with more than 52 individuals.^65,69^ *H. serratifolia* is the only climbing plant that extends to the mature canopy, reaching up to 30 m long, covering 54% of trees in this forest, regardless of support-tree bark type, light requirements, or diameter.^73,74^ This species is shade-tolerant, with intermediate values of area-based photosynthetic capacity (A_max_) and dark respiration (Rd) rates, efficiently intercepts light and reduces gas-exchange rates to cope with the low-light availability found in the mature forest understory.^75^ Furthermore, *H. serratifolia* has registered the highest liana population density among 97 worldwide tropical and temperate mature forests sampled with the same methodology.^65^ *H. serratifolia* presents two phases to attain resources: First, juvenile shoots – by plagiotropic growth – creep along the forest floor in search of a support-tree. They are singular, slender, and reddish, with ventral white adventitious roots ready to attach to a host^76^ and a conspicuous apical greenish bud. The successful juvenile shoots, attached to a tree, climb it by orthotropic growth to access the increased light available in the canopy. At this point, they switch their morphogenesis to the second and adult phase, becoming woody, brownish, and showing reproductive structures (*pers. obs*). The support-tree location pattern of *H. serratifolia*, based on the combination of the juvenile shoot response and spatial variation in forest environmental light, is essential to understand and subsequently link the multiple causes of the abundance, distribution, and dominance of this species in Chilean temperate rainforests.

This study addresses the question: Does the massive woody climber *H. serratifolia* present a support-tree location pattern influenced by light cues in a southern Chilean temperate rainforest? We compared the light received and the direction in two groups of juvenile shoots: Searching shoots (SS), with plagiotropic (creeping) growth versus ascending shoots (AS), with orthotropic growth. We hypothesized that, if low-light availability represents a directional search cue associated with a support-tree location pattern, the light perceived and orientation from the SS group would be similar to those from group AS, exhibiting negative phototropism. The alternative working hypothesis would discard that low-light availability is not a directional search cue nor related to a support-tree location pattern, evidenced by random values of light perceived and orientation in both groups (SS and AS).

## Materials and methods

This study was carried out in the mature forest of Parque Nacional Puyehue (40°39’ S, 72°11’ W, 350 m.a.s.l.), a temperate rainforest at the western foothills of the Andes, in central-southern Chile. Parque Nacional Puyehue exhibits a maritime temperate climate, an annual precipitation of 3500 mm, and minimum and maximum annual average temperatures of 5.4°C and 13.8°C, respectively.^77–79^We set up eight randomly sampling plots of 25 m × 5 m (0.1 ha total) in the forest understory–avoiding gaps, edges or different slopes–, all of them had the same northern facing exposure to prevent possible biases related to different sunlight time exposition.

Inside each plot, we registered the orientation degrees (directionality) of juvenile shoots with plagiotropic (SS = searching shoots group) and orthotropic (AS = ascending shoots group) growth, using a compass (Suunto® A-10, Finland) in relation to the direction of true magnetic north (directional vectors) and the degrees of the sunset across sampling days. For SS, we selected all those juvenile creeping shoots when they pointed to the closest potential support-tree <2.5 m and not to dead ends (e.g., shadow of rocks, dens, or holes in the soil), to avoid possible confounding effects by distance^80^ or a possible automated growth toward darkness. We measured the directional orientation in each juvenile creeping shoot (SS) over its active apical bud. For AS, we defined as orientation reference (direction) the first successful shoot contact point (i.e., starts to climb toward the canopy) at the base of the support-tree trunk, to determine if the path taken on the ground leads it to find the support-tree, not by chance. We chose all those juvenile shoots with orthotropic growth (AS) actively ascending with their adventitious roots attached to the first support-tree 50 cm above the ground (considering this as a measure of successful attachment). SS and AS were measured without differentiation in ramets and genets because it was difficult to identify their origin, although their dynamics and effects in the forest were comparable.^55,81–84^ We did not register tree identity, diameter, and density in potential vs. climbed support-trees because tree stem density has not proven to influence the liana-host selection.^81^ *H. serratifolia* has also shown no support-tree type or diameter preferences.^73,74^ Furthermore, we chose SS and AS because these allow us to compare if there are directional orientation and concordance between the present (SS) and past (AS) information acquiring shoots.^85,86^ SS and AS were expected to access the trunk face that receives less sunlight exposition (southwest, SW), opposite from the trunk facing the sunrise (northeast, NE).

In sampled plots, the light environment on the forest floor and the light radiation received by SS, previously used to register the directional orientation, were quantified using hemispherical photographs. We took a picture 0.3 m above the ground at the center of each plot to characterize the forest light environment. To depict the light availability received by SS, we photographed each creeping shoot just above the active apical bud, as close to the ground as possible (8–10 cm). The photographs were taken with a digital camera (CoolPix 995, Nikon®, Tokyo, Japan), mounted and horizontally leveled on a tripod oriented north to south (by a Suunto® A-10 compass, Finland) with a fisheye lens with a 180° field of view (FCE8, Nikon®). All hemispherical photographs were taken under homogeneous cloudy conditions, near dawn or dusk, to avoid possible bias due to diary natural light fluctuations. The quality of the photos was set at a fine resolution of 1:4 compression in a JPEG format. We calculated, using HemiView® software canopy analysis version 2.1 (2000, Delta-T Devices Ltd, UK) and the geographic location, in each hemispherical photo the indirect site factor (ISF), diffuse proportion of solar radiation expected to reach the point where photographs were taken^87^ and global site factor (GSF), considered as the total amount of diffuse and direct light that penetrates the canopy, reaching a specific point, relative to the amount that would be received with no canopy interception,^88^ analogous to the percentage of photosynthetically active radiation (PAR).^89^

The percentages of diffuse light incidence (ISF %), registered in the forest light environment (inside plots) and above each creeping shoot (SS), were used to compare frequency histograms and density lines to determine whether there were any differences among the values obtained. Likewise, to quantify the light incidence received in the AS, we registered photosynthetic active radiation (PAR; µmol·m^−2^·s^−1^) on the trunk base where each juvenile shoot with orthotropic growth was attached to the support-tree and on the opposite free trunk face (180°) using a LiCor 250A light meter (LiCor, Lincoln, NE, USA). PAR readings on AS were carried out under cloudy conditions at noon, avoiding possible bias produced by the diary natural light fluctuations. At the same time, a full sun PAR measurement was taken outside the forest to convert the light availability of each ascending shoot into the maximum PAR percentage for future comparisons.

All directional data were analyzed with circular statistics,^90–96^ previously used in plant distribution,^97,98^ and Dynophyceae movement.^99^ We assessed whether SS and AS presented: (1) an oriented distribution, (2) differences in their orientation distributions and (3) a relationship between the perceived light availability and the directional search orientation toward the structural support. In SS and AS separately, we carried out the statistical hypotheses test sequence – Kuiper, Watson, and Rayleigh –,^100^ to examine the null hypothesis that SS and AS of *H. serratifolia* are distributed without showing a preferred orientation (uniform distribution, random orientation). These tests can evaluate if the directional data present evidence for uniform, von Mises and unimodal distribution, respectively.^100^ Through the Rayleigh *z* test, due its better performance in comparison with other commonly used tests,^101^ we assessed if the sample data of SS and AS showed a single modal direction,^90,91,100,101^ *z* value represents how large the mean length vector must be to indicate a nonrandom population, with values of more than 0.5 the hypotheses of randomness can be rejected.^102^ The mean resultant length vector *rho* of circular data of SS and AS measures the concentration of unimodal circular data [0, 1]. *rho* values close to 1 denote data strongly clustered around the mean direction (long vector length, reaching the circle border), while values close to 0 indicate data spread more evenly around the circle (short vector length, far from circle border).^93,103,104^ After that, to analyze if the orientation distributions differed significantly between SS and AS, we applied the Watson's two-sample test of homogeneity to compare their directional means.^92,104^

Linear–circular regressions^92,105,106^ were also performed separately for both types of juvenile shoots (SS and AS) to test a possible relationship between shoot direction and the light radiation received. The circular response variable is the directional orientation measured in degrees, and the linear explanatory variable is light availability, quantified by the GSF (%) for SS and PAR (%) for AS (as a meaningful predictor), respectively. A general procedure is not yet available to quantify and measure circular–linear correlations with well-defined properties.^107^ Currently, measures of the hippocampal phase precession weakly depend on prior knowledge of the data^107^ and associations between two circular variables or correlations (with transformations) for linear–linear data analyses.^91,92,105,106,108^ Finally, in AS to compare whether the values of light incidence received (PAR %) differed between the support-tree trunk face where the juvenile *H. serratifolia* shoots were found ascending and the opposite trunk face, we used a paired *t-*test to determine the difference between the two means.^108^

All analyses were conducted in the R software environment 4.2.2,^109^ with a 5% significance level using the “circular” (version 0.4–93),^104^ “CircStats” (version 0.2–6)^110^ and “NPCirc”^111^ packages; including the functions CircularBoxplot,^112^ lm.circular (version 0.4–93)^104^ and kern.reg.lin.circ,^111^ implementing Local-Linear estimators to obtain graphical trend lines.^113^

## Results

**Figure 1.**
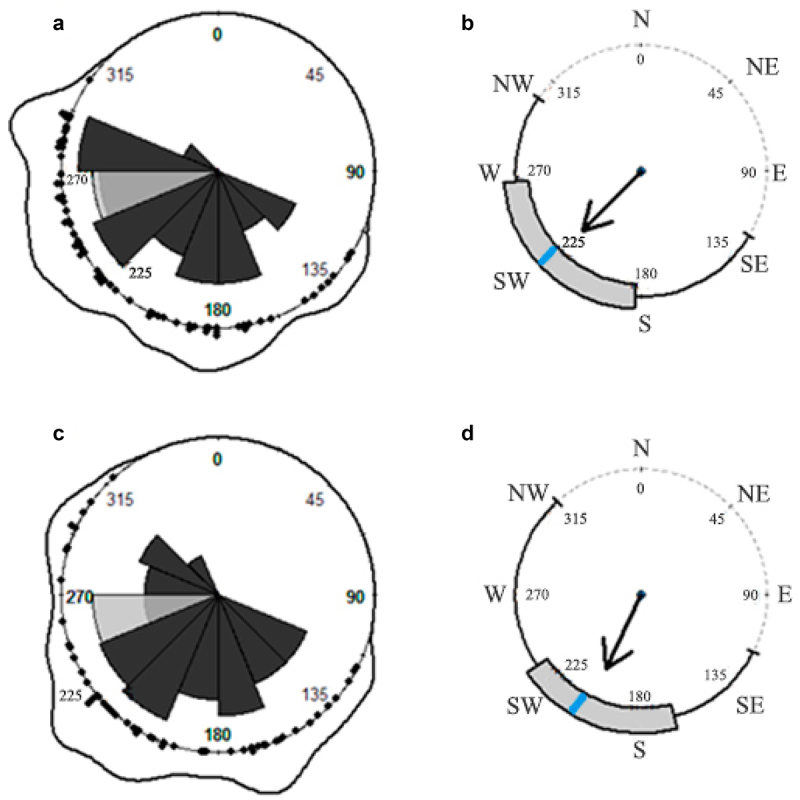
Rose diagrams, density lines and circular boxplots of *H. serratifolia* juvenile searcher (a, b) and ascending (c, d) shoots. Black arrows represent *rho* (pointing to the mean direction) and blue line the circular median value. Light gray petal represents the frequency, in sunset degrees, along the sampling days. Dark gray petals represents the frequency distribution of SS (a) and AS (c), respectively. Black dots represent each individual orientation measurement for SS and AS over the circle (a–c).

*H. serratifolia* searching and ascending shoots presented a significant deviation from a uniform distribution (Figure 1, Table 1). A total of 124 individual of *H. serratifolia* juvenile shoots were found in 0.1 ha. We measured 70 juvenile shoots with plagiotropic (creeping) growth (SS = searching shoots), and 54 ascending on support-trees with orthotropic growth (AS = ascending shoots). The orientation distribution of SS and AS, observed by density lines and rose diagrams, covers the angle range of [135°; 315°] (Figure 1a-1c). The frequency distribution shows that SS and AS share significant frequencies – in orientation degrees – to creep and ascend on the support-tree, among [160°;180] and [225°;250°] (Figure 1a – 1c). In detail, SS on the forest floor exhibited an average directional orientation of 226.2° ± 0.92° (mean ± SD), toward southwest (SW), and an interquartile range of [180°;260°] (Table 1, Figure 1b). AS were oriented, on average, at 205.8° ± 0.90° (mean ± SD); with a tendency to climb on south-southwest (SSW) side of the host tree trunk and an interquartile range of [160°;235°] (Table 1, Figure 1d). Both groups of juvenile shoots tended to creep – or climb – on the support-tree trunk face that receives less sun exposition, considering the frequency in sunset degrees measured along the sampling days (Figure 1a – 1c) and the sun rises in the northeast, NE.

**Table 1.** Circular summary statistics for *H. serratifolia’s* juvenile searcher (SS) and ascending shoots (AS).

Descriptive statistics	Searchers	Ascending
Mean direction (*µ*)	226.2°	205.8°
Median direction	228°	210°
Mean resultant length (*rho*)	0.664	0.653
Circular variance	0.336	0.347
Circular standard deviation (SD)	0.92°	0.90°
Circular dispersion	0.56°	0.57°
Mean direction confidence interval 95%	[211.83°; 236.53°]	[191.03°; 219.91°]

We found using the circular hypotheses test sequence that SS and AS directional data were not uniform distributed (SS *Kuiper’s test*, N = 70, V = 4.414, *p*-value < 0.01; AS *Kuiper’s test*, N = 54, V = 3.475, *p*-value< 0.01), fit the von Mises distribution (SS *Watson’s test*; N = 70, U^2^ = 1.632, *p-*value < 0.01; AS *Watson’s test*, N = 54, U^2^ = 1.219, *p*-value < 0.01), and displayed single directional orientation tendencies (SS *Rayleigh’s uniformity test*, N = 70, *z* = 0.6644, *p*-value = 3.79;^−14^ AS *Rayleigh’s uniformity test*, N = 54, *z* = 0.6535, *p*-value = 9.67 e^−11^). Rayleigh’s uniformity test *z* values for SS (*z* = 0.6644) and AS (*z* = 0.6535) with values of more than 0.5 in the mean length vector indicate that they are nonrandom. Furthermore, no statistically significant differences were found between the mean directions *µ* (orientation) of the two groups of juvenile *H. serratifolia* samples (*Watson’s two-sample test*, N_1_ = 70 SS sample size, N_2_ = 54 AS sample size, critical value = 0.268, U^2^ = 0.1454).

**Figure 2.**
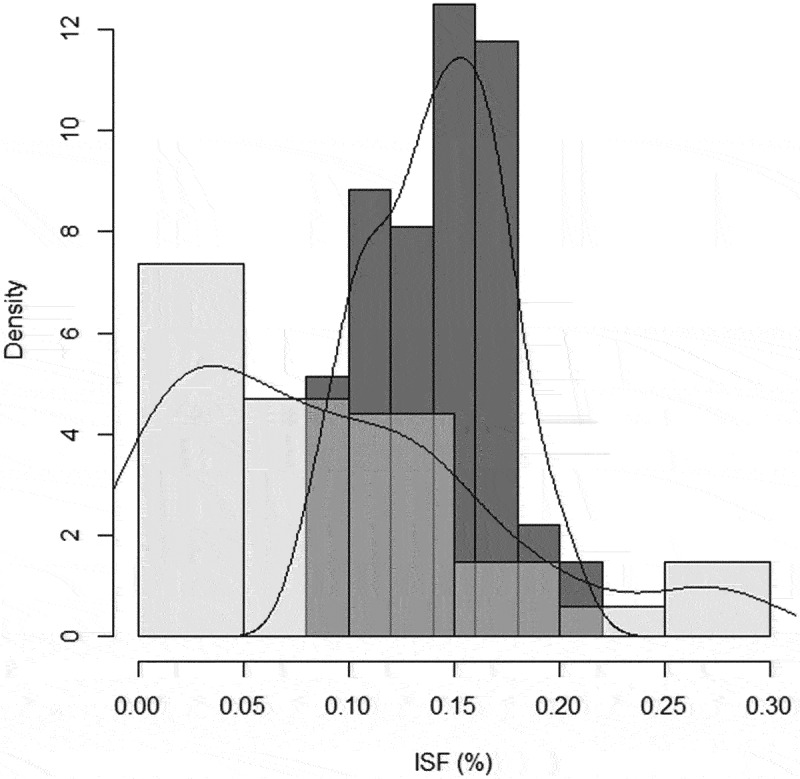
Frequency histograms and density lines of diffuse light incidence (ISF %) registered inside the plots, forest light environment (dark gray bars), and above each SS measured individual (light gray bars).

The linear–circular regressions suggested a possible relationship between directional orientation (circular response variable) and light availability (independent linear predictor) for *H. serratifolia* SS (GSF %, N = 70, *μ*ˆ = −2.04 radians, *κ*ˆ = 1.82 and *γˆ_1_* = – 0.008, S.E. = 0.005, *p*-value *<*0.01; Appendix 1) and AS (PAR %, N = 50, *μ*ˆ = −2.54 radians, *κ*ˆ = 1.83 and *γ*ˆ_1_ = – 0.075, S.E. = 0.039, *p*-value *= *0.028; Appendix 1). The results propose that SS ([7;13] GSF%) and AS ([0.5; 1.5] PAR%) oriented toward the mean direction presented lower light availability values than those oriented away from that direction (Appendix 1). Thereby, the orientation angle of shoots (considering the concentration as well as the mean direction of the distribution of the circular response variable) may be influenced by low values of light availability (linear predictor). Light attenuation influences the directional support-tree pattern of *H. serratifolia* shoots. Furthermore, we found that the diffuse light (ISF %) received by *H. serratifolia* searching shoots (SS) presented a range of lower values ([0; 0.15] ISF %) than those obtained on the forest floor, evidenced by the differences in frequency distributions and density lines between this group and the general forest light environment (Figure 2). Finally, we also found differences between the percentage values of photosynthetic active radiation (PAR %) received by the support-tree trunk face (mean = 1.31) where *H. serratifolia* juvenile shoots were ascending and the opposite (mean = 2.27) free trunk face (paired *t-*test, df = 36.39, *t* = −4.125, *p*-value <0.001).

## Discussion

*H. serratifolia*, a massive and successful woody climber (liana) in Parque Nacional Puyehue, presented a directional pattern of support-tree location based on light attenuation and shoot orientation, with goal-directed biological motion. Searching shoots (SS) were found in reasonably dark sites on the forest floor (Figure 2) and ascending shoots (AS) in very dark sites on support-tree trunks that receive little light exposition. Both groups of juvenile *H. serratifolia* shoots were oriented toward the southwest, with no circular statistical difference among them (Figure 1, Table 1) and a circular–linear relationship between the direction (orientation angles) and light availability perceived (Appendix 1). Support-tree location, including perception and action, requires precision^33–35,46^ and a speed-accuracy trade-off, where rapid and impulsive movements are prone to be more inaccurate than slower and more cautious actions.^114–116^

Climbers are ecological perceivers of possible interactions and key invariant environmental information, such as the opportunity to reach a support that is worth climbing, where perception of affordances and support-tree location mechanisms constitute the climber-support coupled system, considered as a continuous cyclic loop.^33,42,117–123^ In functional terms, plant orientation toward a potential support is similar to an animal running toward its prey,^2,12,122–126^ suggesting the ability to process the features of the support, with a goal-directed and anticipatory behavior.^127^ This study is the first one related to support-tree location by climbing plants in Chilean temperate rainforests.

Negative phototropism, with intrinsic variability while remaining stable and predictable across many climbing plant species, indicates growth or movement away from the light, based on light attenuation and evidenced with a directional pattern of plagiotropic (creeping) shoots and seedlings exposed to bright light, or even to low-light intensity.^38–40,128–130^ This directional pattern can be explained by the inverse square law, which states that light intensity is attenuated in inverse proportion to the square of the distance from the light source.^40,131^ Previous findings related with support-tree location and low-light availability as cue^33,35^ could be considered cases of negative phototropism.^42^ Balcázar et al.^64^ suggested that *Heteropsis* spp. randomly searched for support – without light availability or directional statistical measurements – because their seedlings were found ascending unsuitable support-trees. A random search effort implies pointless plant resource investment^17^; when climbing plants move toward a high light availability, they veer away from support.^44^ Support-trees are a fundamental resource not regularly granted to woody climbers; they provide the darkest sector of the horizon in the chiaroscuro of the forest floor.^33^ Negative phototropism, as an active response involving acquiring and processing information (light discrimination), allows climbing plants to be attracted and reach the darkest side of the support-tree base, to ascend toward the forest canopy.^38^ We found in *H. serratifolia* juvenile shoots that low-light availability represents a search cue associated with a directional support-tree location pattern based on light attenuation (Appendix 1), exhibiting negative phototropism. The orientation and light received by searching (SS) and ascending (AS) shoots of *H. serratifolia* are similar between them (Figure 1, Table 1). These outcomes support our alternative work hypothesis and discard the fact that *H. serratifolia* juvenile searching (SS) and ascending (AS) shoots randomly search for support-trees. Negative phototropism greatly influences a shoot’s ability to reach a support-tree. Furthermore, *H. serratifolia* juvenile shoots based on our work hypothesis, the selection of those individuals toward some potential support-tree and not toward dead ends, and the results obtained express the possibility that this plant present light discrimination, as it has been reported in previous studies^132–134^ and may be a more refined process of perception and action, considered as cognitive behavior.^2,127,135^

Support-tree location patterns of lianas (woody climbers) have been documented mainly in tropical,^33,35,51,64,83,84^ north temperate or subtropical forests.^81,117,118,136–140^ Factors including the characteristics of the support-tree, its proximity, and availability all strongly influence the selection by climbing plants.^33,47,81,83,141–146^ Also, on the forest floor, under low-light availability, when *H. serratifolia* shoots do not find a host tree, they have been observed as transient erect shoots, as has been the case of *Hydrangea petiolaris*, which plays the role of a seedling bank,^147^ thriving near the survival limit by its shade tolerance.^148^ Our study, in a southern temperate rainforest, presents the inherent limitation of not controlling all the variables, including the microclimate (*e.g*., wind, moisture) and ecological factors (*e.g*., herbivory, competition) that could influence the circular distribution along with natural local scale orientation patterns.^98,103^ Plants can sense several cues, biotic and abiotic vectors, including electrical, magnetic, or volatile organic compounds.^2,18,149,150^ This means that noise must be added to the relationship between light and direction accuracy. The support-tree location pattern presented in this survey is not enough to explain the dominance of *H. serratifoila*, though it is an important contribution to understand its natural history. A probable answer to explain the dominance of *H. serratifoila* in this temperate rainforest could be based on its characteristic traits set like infestation rate, shade tolerance, adventitious root climber, clonal nonrandom foraging, and exhibition of negative phototropism (as light discrimination), that could confer an environmental advantage over other coexisting climbing plant species in this temperate rainforest, due the high abundance of support-trees in this forest.^74^

Clonal growth in climbing plants allows plagiotropic shoots to spread horizontally and influences the nonrandom foraging behavior for resources.^4,51,54,55,151–153^ This anticipatory behavior, and clonal ability, incorporates epigenetic memory of past, present information and environmental interactions.^85,154–156^ Shoot typologies aid in understanding the growth and branching patterns of woody climber stems, according to their dynamics in time and space.^51^ If only present information prevailed, with no input from acquired memory, every juvenile shoot of *H. serratifolia* would start from scratch in the development of strategies to acquire its necessary resources; then the orientations of ascending and searching shoots of *H. serratifolia* should have differed in each growing season. An eternal and generational error-loop. The light discrimination, as negative phototropism exhibited by *H. serratifolia* juvenile shoots, could be a demonstration of anticipatory behavior, showing that searching shoots are not attracted to any dark site in the chiaroscuro of the forest floor, they are attracted to shadows in the understory to find a lighter environment like the canopy. However, studies in the climbing plant species of this temperate rainforest are still lacking when it comes to clonal genetic diversity, population structure,^157–159^ physiological shoots integration,^160–162^ community clonal growth and foraging.^163–166^

Plant behavior,^6,167–169^ intelligence^11,123,149,167,169,170^ and cognition^2,171^ concepts have been discussed metaphorically and literally.^44,45,122,126,150,172^ Nonetheless, a prevailing misconstruction is that a precise scientific study is not viable without an unambiguous definition, even though the definition and the field of research could evolve together.^168,173^ The search for semantic purity in plant behavior has encouraged the tendency to reject important concepts and results in behavioral ecology, in the framework of ultimate causation.^122,126,167,168,172^ Plant physiology cannot explain plant behavior before a phenomenon appears. It is far more interesting and relevant to understand how plants live under a plural approach, discovering morpho-physiological, cognitive processes and ecological causes and consequences of their sensory systems. This broad approach allows us to understand how the transmission and integration of cues occur in real time, leading to survival and adaptation in ever-changing environments.^174–176^ Sensory biology is an emerging frontier in the study of the growth and functioning of climbing plants^177^ and the experimental test of the ocelli-based *plant vision*^132–134^ would be the logical next step in our quest for understanding the plant sensory complexity. Future studies in *H. serratifolia* need to take into account the possibilities that (1) negative phototropism combined with the effect of gravitropism could guide and refine the growth in these plants and (2) how in physiological and behavior terms, *H. serratifolia* twin around toward the sunniest part of the support-tree and the concomitant switch in morphogenesis from juvenile to adult phase.

The selective foraging behavior response and resource acquisition pattern observed in *H. serratifolia* reflect its ability to adaptively assess, adjust and construct flexible information processes in ecological time, optimizing its performance in unpredictable and dynamic environments.^2,7–11,170,178,179^ The genome level, with heritable variation, produces morphology and physiology, which sequentially guided by sensory information, develops behavior, and this, in turn, generates the fitness that selection can act upon.^180^ Behavior is a morpho-physiological, inescapable sensory-guided consequence of natural selection, acting upon any living being, with an evolutionary function as an adaptive mechanism; a causal link exists between fitness and sensory systems.^1–3,172,181,182^ *H. serratifolia* explores the chiaroscuro on the forest floor, where low-light availability represents specific and anticipatory information regarding a potential support-tree (causal pattern), due to the inherent, invariant, and interactive climber-support coupled system. Negative phototropism, as light discrimination, provides and represents a parsimonious influence to guide the shoots of *H. serratifolia* toward the support-tree in the forest, cues of low-light availability (“how”), its direction (“where”), and encoding spatial and temporal (“when”) information in the heterogeneous light environment.

## Supplementary Material

Supplemental Material

## Data Availability

All data and/or R codes that support the results of this survey are available from the corresponding author upon reasonable request.

## Geolocation information

The study location is “Parque Nacional Puyehue”, 40°39’ S/72°11’ W, central-southern Chile, at the western foothills of the Andes.
