# Supplementary material for: From dark to darkness, negative phototropism influences the support-tree location of the massive woody climber Hydrangea serratifolia (Hydrangeaceae) in a Chilean temperate rainforest
Source: Plant Signal Behav. 2022 Dec 7;17(1):2122244. doi: 10.1080/15592324.2022.2122244 (PMC9733698; doi:10.1080/15592324.2022.2122244)
Supplement: Supplemental Material [file KPSB_A_2122244_SM6761.docx]

***Supplementary material.*** Photographs of searching (SS: SS1, SS2) and ascending (AS1, AS2, AS3) shoots. SS1 show evasion of dead ends (e.g., piece of wood) on the forest floor. SS2 show a coexisting species *Boquila trifoliolata* on the forest floor. AS group pictures show how the PAR measurements were made at the most shaded part of the support-tree base, as starting point to climb ascending to the canopy.
